# Rate of Leiomyosarcomas during Surgery for Uterine Fibroids: 8-Year Experience of a Single Center

**DOI:** 10.3390/jcm12247555

**Published:** 2023-12-07

**Authors:** Dimitrios Zouzoulas, Dimitrios Tsolakidis, Olga I. Pavlidi, Panagiotis D. Pappas, Theodoros Theodoridis, George Pados, Stavroula Pervana, Elsa Pazarli, Grigoris Grimbizis

**Affiliations:** 1 1st Department of Obstetrics & Gynecology, “Papageorgiou” Hospital, Aristotle University of Thessaloniki, 56403 Thessaloniki, Greecepavlidihr@gmail.com (O.I.P.); pappan1986@gmail.com (P.D.P.); theodtheo@yahoo.gr (T.T.); padosgyn@gmail.com (G.P.); grigoris.grimbizis@gmail.com (G.G.); 2 Anatomical Pathology Laboratory, “Papageorgiou” Hospital, 56403 Thessaloniki, Greece

**Keywords:** leiomyosarcoma, morcellation, fibroid

## Abstract

The aim of this study is to investigate the prevalence of occult malignant mesenchymal tumors in patients operated on for uterine fibroids in relation to the surgical approach and type of operation. A retrospective review of all patients that underwent surgery for uterine fibroids (January 2011–December 2018) at the 1st Department of Obstetrics & Gynecology at “Papageorgiou” Hospital. The surgical approach and clinicopathological characteristics were analyzed. A total of 803 patients were operated on: 603 (75.1%) with laparotomy, 187 (23.3%) laparoscopically, and 13 (1.6%) vaginally. Furthermore, 423 (52.7%) patients underwent hysterectomy and 380 (47.3%) myomectomies. Laparoscopy and myomectomy were offered to younger patients with fewer smaller uterine fibroids and were associated with statistically significant shorter hospitalization. The pathological reports revealed: 690 (86%) benign leiomyomas, 32 (4%) cellular leiomyomas, 29 (3.6%) degenerated leiomyomas, 22 (2.7%) adenomyomas, 18 (2.2%) atypical-bizarre leiomyomas, 1 (0.1%) STUMP, 5 (0.65%) endometrial stromal sarcomas, and 6 (0.75%) cases of leiomyosarcomas (LMS). All LMS were preoperatively characterized as suspicious and underwent abdominal hysterectomy. Morcellation was offered in two cases of atypical leiomyomas, with no morcellation-associated complication. Laparoscopy as a valuable surgical approach for young patients with fewer in number and smaller in size fibroids is associated with shorter hospitalization. The risk of unintended morcellation of LMS seems to be very low and can be reduced with careful preoperative work-up but not eliminated.

## 1. Introduction

Leiomyoma is the most common neoplasm of the female genital tract and arises from the uterine smooth muscle. It can appear at any place topographically in the uterus and it is categorized based on orientation and location. It is important to correctly identify the position of the fibroid, corelate it to possible symptoms, and choose the suitable treatment plan. However, there were discrepancies between physicians in the fibroid topography report. This matter was resolved by the new FIGO classification system about fibroids, which is much more detailed [1]. It retains the original submucosal relationship of types 0–2 but extends staging to an additional six categories. Type 3 fibroids are near the endometrium but are completely intramural. Type 4 describes a completely intramural fibroid; types 5 and 6 are defined by the relationship to the serosal layer; type 7 describes fibroids that are pedunculated on the sub-serosal surface; and type 8 refers to fibroids found in ectopic locations such as the cervix [2].

Another important factor in the identification of the fibroids is the final pathology report. Taking into consideration their microscopic pathologic features, known as Stanford criteria [3], they can be divided into four groups: (1) benign leiomyomas, (2) benign variants of leiomyomas (e.g., cellular or bizarre), (3) smooth muscle tumor of uncertain malignant potential (STUMP), and (4) sarcomas [4]. Sarcomas can be further categorized into: (a) leiomyosarcomas, (b) endometrial stromal sarcomas, and (c) undifferentiated sarcomas [5]. The Stanford criteria help to differentiate between malignant and benign variants of the fibroids based on the following criteria: (1) tumor cell necrosis (not infract necrosis), (2) diffuse moderate to severe cytological atypia, (3) increased mitotic activity (a mitotic count of at least 10 mitotic figures (MF)/10 high power fields (HPFs)). In order to establish the histologic diagnosis of a leiomyosarcoma, at least two criteria must be present, while for STUMP only tumor cell necrosis must be present with no other criteria and for bizarre only cytological atypia. Discrimination between these histological features is quite challenging and many times intraobserver variability is reported between pathologists [6].

Leiomyomas or fibroids have a higher incidence compared to the other aforementioned tumors, which is 70% for white and over 80% for Black women in their fifth decade [7]. There are some risk factors that are linked with increased incidence of uterine fibroids: early menarche, nulliparity, obesity, late entry into menopause, and positive family history of uterine fibroids. But, there is also a decreased risk of uterine fibroid presentation with increased parity, late menarche, smoking, and use of oral contraceptives [8]. Even though they are benign, they can have a significant impact on the everyday physical and mental well-being of women with this condition. They can arise from different parts of the myometrium and their growth is primarily hormone-dependent, based on the circulating estrogen levels [9]. However, further knowledge about their pathogenesis is not well understood, but research suggest that it begins with a single uterine smooth muscle cell (myometrium) which is then followed by deviations from the normal signaling pathways of cellular division. They can be asymptomatic, but often they cause prominent symptoms, such as heavy menstrual bleeding or abdominal/pelvic discomfort [10], which lead to treatment. This treatment plan could include either surgical therapy or medical management. Surgical therapy includes hysterectomy, myomectomy (fertility preservation), MRI-guided focused ultrasound surgery (focused ultrasounds waves resulting in cauterization), uterine artery embolization (fertility preservation), and endometrial ablation (when the primary symptom is heavy or abnormal uterine bleeding). Alternatively, medical management focuses on a decrease in symptoms and includes oral hormonal contraceptives, a levonorgestrel intrauterine device (IUD), gonadotropin-releasing hormone (GnRH) agonists, and nonsteroid anti-inflammatory drugs (NSAIDs).

On the other hand, LMS are rare and represent 1% of all uterine malignancies but are still the most common histopathologic type of uterine sarcomas [11]. They usually affect postmenopausal women (around 55 years old) and are presented as solitary, large (mean diameter of 10 cm) tumors, located intramurally with infiltrating or irregular margins and a soft/fleshy cutting surface, compared to multiple, regular-shaped fibroids with a cut surface that is usually soft and fleshy with alterations of necrosis and hemorrhage [4]. It is unlikely that LMS arise from pre-existing leiomyomas and most investigators believe that they are sporadic and arise de novo [12]. Recent studies in molecular pathology have shown that miRNA profiles between LMS and leiomyomas are completely different [13], supporting the above-mentioned assumption. However, the presence of leiomyoma-like areas in LMS (specifically, cellular and symplastic areas) with the discovery of additional genetic aberrations in the sarcoma, differences in immunohistochemical profiles between the benign and sarcomatous areas within the same tumor, and demonstration of identical patterns of X-chromosome inactivation between benign and malignant tumors suggest that a minority of tumors indeed originate from pre-existing leiomyomas [14,15,16]. Uterine LMS are classified into different histologic subtypes, based on cellular characteristics and constituents of the intercellular stroma [17]. Most are of the spindle cell type (usual differentiation) and leiomyosarcomas with epithelioid or myxoid differentiations are less common. Unfortunately, preoperative differential diagnosis between LMS and leiomyomas is elusive because no reliable tool exists and typically they present with symptoms almost identical to ordinary fibroids.

These smooth muscle tumors remain the leading indication for hysterectomy worldwide annually [8]. Their surgical treatment includes myomectomies for young women who wish to preserve their fertility or hysterectomies for women who have completed their family planning. Depending on their location and the surgeon’s skills, the surgical approach varies from laparotomy to minimal invasive surgery (MIS) (laparoscopy or robotic surgery), as well as vaginal surgery or hysteroscopy [18]. Studies have shown that laparoscopic myomectomies compared to laparotomy have better results concerning morbidity and hospital stay, thus establishing them as the treatment of choice for premenopausal women [19]. Furthermore, power morcellation was approved by the Food and Drug Administration (FDA) in 1995 as a primary method used for the removal of uterine fibroids during laparoscopic myomectomy [20]. By this procedure, electrical energy is transformed into mechanical power, cutting uteri or fibroids into smaller pieces for removal from the abdominal cavity via a 12–20 mm ancillary port [21]. The use of power morcellation as the method of choice to remove the uterine neoplasms has become a controversial issue in USA and Europe in the last decade due to a risk of unintended morcellation of LMS [22]. This could lead to the spread of cancerous cells to the whole abdominal cavity [23], resulting in poorer prognosis of these patients. This led to an FDA warning in 2014 discouraging MIS and power morcellation for the removal of leiomyomas. Firstly, the FDA statement divided patients who should not undergo morcellation in two groups: patients who are in “peri or post-menopausal” states and/or who are candidates for “en bloc tissue removal” [24]. Later, the FDA required the use of a morcellator with a containment system or advised special controls [25]. These final FDA orders created a bewilderment and perplexity in the world gynecological community worldwide and resulted in an increase in laparotomy as a surgical approach for fibroids, followed by an increase in postoperative complications [26] and total costs per patient [27].

The aim of this study is to identify the true rate of occult LMS in patients operated on for presumed uterine fibroids regarding the surgical approach and the type of operation. We believe that the prevalence is much lower than presented in the literature, for any age group, and that careful pre-operative work-up can detect these suspicious cases.

## 2. Materials and Methods

### 2.1. Patient Eligibility Criteria

After acceptance of the study from the hospital’s ethical committee, the medical records of all patients (*n* = 914) that underwent surgery with the indication of uterine fibroids from January 2011 to December 2018 at the 1st Department of Obstetrics & Gynecology at “Papageorgiou” Hospital of Thessaloniki were retrospectively reviewed. According to the inclusion and exclusion criteria that were set for this retrospective study, we identified 803 patients eligible for further analysis.

Inclusion criteria:

- Preoperative confirmation that the tumor arises from the uterus;
- Preoperative transvaginal ultrasound, laboratory exams, and detailed medical history;
- Surgical treatment should include myomectomy or hysterectomy;
- Informed consent signing.

Exclusion criteria:

- Prior malignancy of the female genital tract;
- Mesenchymal tumor located in other pelvic organs (e.g., ovarian fibromas);
- Missing important data (e.g., histology).

### 2.2. Study Design

The pathology reports of the selected patients (*n* = 803) were extensively studied to identify and categorize the type of smooth cell tumors based on their microscopic morphological features. The criteria for the diagnosis of malignant or benign variants were (1) cytological atypia, (2) increased mitotic activity (>10/10 hpf), and (3) tumor cell necrosis (not infract necrosis). LMS need to meet at least two of the above-mentioned criteria, STUMP present with tumor cell necrosis and no other criteria, while Bizarre leiomyomas present only cytological atypia. When the pathological report was not clear (*n* = 53) for the three above-mentioned diagnostic criteria, two expert pathologists independently reviewed the relevant pathological slides in order to establish a final diagnosis.

All patients underwent either myomectomy (fertility sparing surgery) or hysterectomy and the surgical approach was laparotomy, laparoscopy (since robotic surgery is not available in our institution), or vaginal surgery. Data selection included age, hospital stay, and specific information about the uterine fibroids: histological type, number (multiple or solitary), diameter, location (submucosal, intramural, subserosal, pedunculated), and orientation (posterior, anterior, lateral, fundal).

The analysis of the selected data was based on the surgical approach and the type of operation. Firstly, they were divided into three groups according to the surgical approach: Group A included women treated laparoscopically, Group B were women treated with laparotomy, and Group C were women that underwent vaginal surgery. Secondly, according to the type of operation (excluding vaginal surgery due to its low incidence), they were categorized into two groups: Group A included women that underwent myomectomy and Group B women that were offered hysterectomy. All the aforementioned data were statistically analyzed between the groups and then further analysis was conducted for the incidence of LMS cases.

### 2.3. Statistics

Statistical analysis was performed using RStudio. Continuous variables were checked for normality and parametric or nonparametric tests were applied, accordingly. Moreover, categorical variables were presented as counts and percentages. Chi-squared or Fisher exact tests were used for the comparison between the two groups. All results were rounded to one decimal. Statistical significance was set at *p*-value < 0.05.

## 3. Results

A total of 803 patients that were operated on, with the indication of uterine fibroid, met the inclusion and exclusion criteria of this study. The mean patient age was 44.85 ± 8.85 years old. Overall, 2760 uterine smooth cell tumors were removed. In total, 377 (47%) patients presented with solitary and 426 (53%) with multiple uterine tumors. Concerning the topography of the fibroids, they were categorized based on their location and orientation. In multiple fibroids, the largest in diameter was considered for the below-mentioned classification. The most common location was intramural, 478 (59.5%) cases, followed by subserosal, 98 (12.2%), submucosal, 76 (9.5%), pedunculated, 29 (3.6%), and intraligamentous, 18 (2.2%). On the other hand, they were almost equally distributed as anterior, 106 (13.2%), posterior, 96 (12%), fundal, 113 (14.1%), or lateral, 38 (4.7%), based on their orientation. However, there were missing data of location in 104 (13%) cases and of orientation in almost half of the cases, 450 (56%). Clinical characteristics of the uterine fibroids are described in Table 1.

After reviewing all the pathology reports by the authors and by the two expert pathologists, in the cases that the three previous mentioned diagnostic criteria were not clearly described in the final report, 690 (86%) benign leiomyomas, 32 (4%) cellular leiomyomas, 29 (3.6%) degenerated leiomyomas, 22 (2.7%) adenomyomas, 18 (2.2%) atypical-bizarre leiomyomas, 1 (0.1%) STUMP, 5 (0.65%) endometrial stromal sarcomas, and 6 (0.75%) cases of leiomyosarcomas were revealed. The histopathological features of the presumed benign fibroids in the initial diagnosis are presented in Table 2.

Furthermore, patients were initially analyzed based on the surgical approach that was offered to them. In total, 187 (23.3%) of them were treated by laparoscopy, 603 (75.1%) by laparotomy, and 13 (1.6%) underwent vaginal surgery, mainly due to concurrent prolapse of the uterus. The age of the patients, total hospitalization, size (larger one, when multiple were present), and total number of the fibroids were analyzed. Statistical analysis between the three groups showed that laparoscopy was offered to younger patients with fewer and smaller uterine fibroids and was associated with statistically significant shorter hospital stay. The aforementioned data are presented in Table 3.

Moreover, we divided patients based on the type of surgery and then we further analyzed it with the surgical approach while looking at the final histopathological report. We excluded the vaginal surgery group from this analysis because we would not be able to draw any important conclusion due to its low number of patients. In total, 423 (52.7%) patients were offered a hysterectomy and 380 (47.3%) a myomectomy.

The myomectomy group had statistically significant younger patients, fewer and smaller uterine fibroids, with also a shorter hospital stay. Unfortunately, two patients with a non-benign histopathology were offered laparoscopic myomectomy with power morcellation and the final pathology report revealed one bizarre leiomyoma and one STUMP. There were also two cases of bizarre leiomyomas that were offered an open myomectomy (laparotomy). No morcellation-associated complications were reported and no recurrences were documented during the follow-up of the patients and, especially, the two patients (with bizarre and STUMP leiomyomas) that underwent morcellation are alive with no evidence of disease. On the other hand, in the hysterectomy group, a total of twenty-five cases of potential malignant and malignant uterine tumors were identified in the final pathology report: fourteen cases of bizarre leiomyomas, five of endometrial stromal sarcomas, and six of leiomyosarcomas. Remarkably, they were all treated with open hysterectomy (laparotomy) because pre-operative work-up characterized these tumors as suspicious. Table 4 shows the relevant data of the myomectomy group and Table 5 of the hysterectomy group.

Last but not least, additional analysis was performed in the LMS that were identified in the final pathology report since this was the aim of this study. The mean age of women with LMS was 65.83 ± 13.54 years old, the mean diameter of the tumor was 104 ± 52 mm, and all presented as solitary “fibroids”. Four cases were grade 3 and two were grade 2 LMS. All LMS cases were preoperatively characterized as fibroids with some “suspicious” features and underwent abdominal hysterectomy (laparotomy). So, the prevalence of occult LMS during morcellation was 0%. Follow-up of these patients showed that all of them relapsed within the first year and that only one patient remains alive until now. This supports the idea that the poor prognosis of these patients is related to the disease itself and not to the surgical approach that was chosen. Table 6 describes the survival data of the LMS cases.

## 4. Discussion

Laparoscopy was regarded as the surgical approach of choice for the treatment of fibroids. However, the potential risks with the use of morcellators in order to remove the tissue specimens away from the abdominal cavity are (1) direct injuries to other organs, (2) parasitic leiomyomas in the peritoneal cavity, and (3) spread of cancerous tissue in the peritoneal cavity due to occult malignancies [23,28]. Those risks changed the indication in the later years and there was noticed a shift from laparoscopy to laparotomy in daily practice, even though there are positive studies for morcellation [29]. This caused an ongoing debate concerning the oncological safety of laparoscopic myomectomy. The aim of this study was to identify the true rate of occult LMS in patients operated on for presumed uterine fibroids regarding the surgical approach and the type of operation.

We investigated which surgical approach was offered to the patients undergoing surgery with the indication of uterine fibroids. The majority of the patients (75%) were offered laparotomy, which is in concordance with the clinical practice that is followed worldwide after the FDA statement in 2014, even though many centers worldwide have opposed to this change from MIS to open surgery. Our data show that laparoscopy was offered to younger patients with fewer and smaller uterine fibroids and was associated with a statistically significant shorter hospital stay, which indicates the superiority of MIS for young patients that want to preserve their fertility. Moreover, we analyzed the type of surgery that was offered to the patients with the indication of uterine fibroids. The myomectomy group, which is of interest due to the morcellation needed during laparoscopy, had statistically significant younger patients, fewer and smaller uterine fibroids, with also a shorter hospital stay.

Interestingly, half of the patients in the myomectomy group underwent laparoscopy with morcellation, but in the final pathology report no LMS or other malignancy was found. There were only two cases of potential malignant tumors: one bizarre leiomyoma and one STUMP. All cases of LMS or other types of sarcomas were pre-operatively characterized as uterine fibroids with suspicious characteristics and they were treated with open hysterectomy. The overall prevalence of LMS in our study was 0.75%, which is one of the highest compared to other studies in the literature, like a large retrospective study with 4791 women from a teaching hospital in Norway with a rate of 0.54%. We believe that this result is explained by the fact that we are a university teaching hospital which is well-recognized as a center for gynecological malignancies and advanced laparoscopic surgery, so all suspicious cases are referred to our clinic. On the other hand, the rate of occult LMS during laparoscopic myomectomy and morcellation is 0%, which is in accordance with two large retrospective studies, which demonstrated a rate of 0% [30] and 0.02% [31], and a recent review [32], with a prevalence of 0.07%. However, our data disagree with the published results by the FDA in 2014 and from another retrospective study including 921 patients with a higher rate of 0.2% of LMS [18].

Our original hypothesis that the prevalence of occult LMS is much lower than the presented in the literature was verified by our results, even though the overall LMS rate was high (0.75%). This is a result of a careful pre-operative work-up in order to identify suspicious cases. Our pre-operative plan consists of three main pillars: (1) detailed medical history, (2) imaging (ultrasound, magnetic resonance imaging), and (3) laboratory exams (CA-125, LDH, LDH isoenzymes). Firstly, the detailed medical history identifies the possible risk factors, such as age, menopausal status, fibroid size and/or rapid growth, history of tamoxifen or pelvic radiation, and hereditary conditions (e.g., Lynch syndrome) [33]. Secondly, concerning imaging, the exam of choice is ultrasound combined with Doppler (by an experienced IOTA certified sonographer) and if a suspicious fibroid is identified, then a magnetic resonance imaging (MRI) is performed. Studies failed to show any superiority between the two modalities [5], so due to cost-effectiveness MRI is considered as a second-line imaging technique. Ultrasound findings that indicate a possible sarcoma are large or rapidly growing, unique heterogeneous ill-defined tumors with irregular distribution of vessels and a low resistance index but high systolic velocities (color score 3–4) [34]. With MRI (T1-weighted images), sarcomas show a heterogeneous hypointensity with hemorrhagic areas, presenting heterogeneous enhancement, after contrast administration, due to areas of necrosis and hemorrhage [35]. The use of LDH and LDH isoenzymes can be useful for the identification of LMS, especially in combination with MRI [36].

When we further analyzed the six cases of LMS that were pre-operatively identified as suspicious and underwent open hysterectomy. We discovered that all cases relapsed within the first year of the surgery and that only one patient is still alive with evidence of disease, while most cases were grade 3. These findings strengthen the assumption that the poor prognosis of the patients with LMS is based on the pathophysiology of the disease and not on morcellation [37]. Leiomyosarcomas metastasize hematogenously rather than by direct extension, so it is unclear whether peritoneal dissemination is the only factor responsible for adverse outcomes. Thus, dissemination of the tumor throughout the peritoneum may not necessarily increase true tumor burden. It may be that any type of tumor penetration at the time of surgery will enhance the hematogenous spread of tumor cells, meaning that tumor spread may occur prior to and independent of morcellation [10]. This is in agreement with the findings of a recent review, which states that disease-free survival is not affected by morcellation and that prognosis is influenced only by tumor size and grade [38]. So, particularly for early stage LMS, morcellation compared to en block uterine removal results in a higher risk of tumor dissemination and recurrence and poorer overall survival [10]. On the other hand, some studies did not find a statistically significant difference in disease-free survival [39,40]. We should keep in mind that it is hard to extrapolate safe conclusions because of the heterogeneity, the small numbers, and the retrospective nature of the various studies in the literature.

Last but not least, it is of high importance to state that even in centers with a very low rate of occult LMS during laparoscopic myomectomy and morcellation, patients should be individually informed about the possible risks and benefits of open and laparoscopic myomectomy/hysterectomy and especially the detrimental results of morcellation in cases with LMS. Clear pre-operative informed consent should be taken and kept within the patient’s medical records. Furthermore, a promising technique to overcome the drawbacks of malignant tissue spread within the peritoneal cavity is to perform leiomyoma morcellation in a bag. However, it is important to keep in mind that there are no data to support myomectomy with in-bag morcellation in suspicious uterine fibroids for malignancy. Further studies are needed to establish its effectiveness and safety for LMS cases [41] despite the fact that the majority of studies have shown comparable results in terms of surgery duration and blood loss [42].

The main limitation of our study is its retrospective nature and that it expands almost a decade back in time. Moreover, there are a lack of demographic data for the total population, but important data for the uterine fibroids were collected. On the other hand, our study features some key strengths which are its relatively large sample size and the low percentage of missing values in our database. Also, the most important fact was the review the Stanford criteria by two expert pathologists when the final histology report was unclear on this matter.

## Figures and Tables

**Table 1:** Characteristics of patients with uterine fibroids.

	Number	%
**Fibroids per woman**		
Solidary	377	47
Multiple	426	53
**Location**		
Intramural	478	59.5
Subserosal	98	12.2
Submucosal	76	9.5
Pedunculated	29	3.6
Ligament	28	2.2
Missing data	104	13
**Orientation**		
Anterior	106	13.2
Posterior	96	12
Fundal	113	14.1
Lateral	38	4.7
Missing data	450	56

**Table 2:** Histopathological features of initially presumed benign fibroids.

	Number	%
Benign leiomyomas	690	86
Cellular leiomyomas	32	4
Degenerated leiomyomas	29	3.6
Adenomyomas	22	2.7
Atypical-bizarre leiomyomas	18	2.2
STUMP	1	0.1
Endometrial stromal sarcomas	5	0.65
Leiomyosarcomas	6	0.75

**Table 3:** Categorization based on the surgical approach.

	Laparoscopy	Laparotomy	Vaginal	*p*-Value
Number	187 (23.3%)	603 (75.1%)	13 (1.6%)	-
Age (years)	38.5 ± 7.1	46.7 ± 8.4	51.8 ± 7.3	<0.05
Hospitalization (days)	2.7 ± 1	4.9 ± 1.8	4 ± 2.3	<0.05
Size (mm)	60 ± 24	77 ± 43	62 ± 24	<0.05
Fibroids per woman	2.9 ± 3	3.6 ± 3.6	3.2 ± 3.3	<0.05

**Table 4:** Myomectomy group.

	Laparoscopy	Laparotomy	*p*-Value
Number	179 (47.1%)	201 (52.9%)	-
Age (years)	38 ± 6.8	40.1 ± 5.5	<0.05
Size	60 ± 24	76 ± 34	<0.05
Fibroids per women	2.7 ± 2.9	3.1 ± 3.5	0.1193
Atypical-bizarre leiomyomas	1	2	-
STUMP	1	0	-
Leiomyosarcomas	0	0	-

**Table 5:** Hysterectomy group.

	Laparoscopy	Laparotomy	*p*-Value
Number	8 (2%)	402 (98%)	-
Age (years)	49 ± 5	50.1 ± 7.6	0.8115
Size	64 ± 27	78 ± 47	0.4316
Fibroids per women	6.1 ± 4.3	3.9 ± 3.7	0.1315
Atypical-bizarre leiomyomas	0	14	-
Endometrial stromal sarcomas	0	5	-
Leiomyosarcomas	0	6	-

**Table 6:** LMS disease-free (DFS) and overall (OS) survival.

	Recurrence	DFS (Months)	Alive	OS (Months)
1st	Hepatic (parenchymal)	6	No	18
2nd	Abdominal wall	10	Yes	95
3rd	Peritoneal carcinomatosis	6	No	14
4th	Pelvic lymph nodes	3	No	20
5th	Ascites	4	No	6
6th	Peritoneal carcinomatosis	7	No	16

## Data Availability

Data are available after request in excel files.
